# Post-TAVI outcomes: devil lies in the details

**DOI:** 10.18632/aging.102382

**Published:** 2019-11-13

**Authors:** Ignacio J. Amat-Santos, Pablo Díez-Villanueva, Javier López Diaz

**Affiliations:** 1 CIBERCV, Cardiology Department, Hospital Clínico Universitario, Valladolid, Spain; 2 Cardiology Department, Hospital Universitario La Princesa, Madrid, Spain

**Keywords:** renin-angiotensin system, aortic stenosis, ventricular remodeling, poli-medication, TAVR

The current population treated with transcatheter aortic valve implantation (TAVI) conform a cohort of advanced age patients [1]. In particular, in those with recommendation for TAVI according to the guidelines [2], namely patients considered of intermediate and high risk for open surgery, all the evidence is based on studies including patients aged beyond 75 years. However, more recently the results of this therapy have also been found better than surgery in lower risk patients through several studies that specifically included patients below 70 years of age [3]. In such population, prosthesis durability was the most important concern given their longer life-expectancy. However, as longer-term follow up is available for this technology, durability seems comparable or even better than that of surgical bioprosthesis thanks to better hemodynamics of transcatheter devices [4].

**Figure 1:**
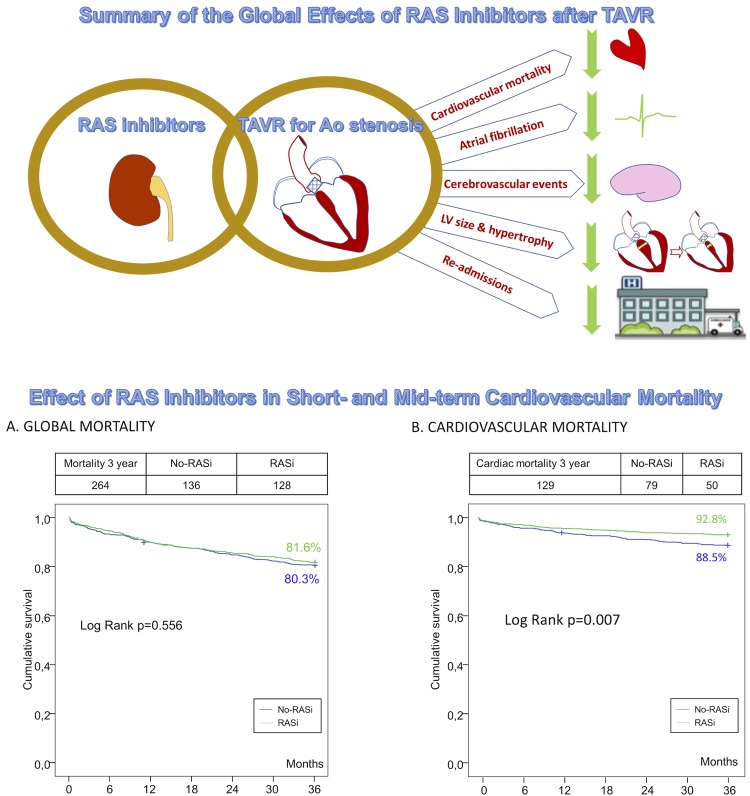
Summary of the global effects of RAS inhibitors after TAVR.

With such positive finding, the current major concern has shifted to the limited evidence on post-procedural pharmacological aspects, often treated as mere detail, but that can equally impact long-term outcomes after TAVI. The management of anticoagulation, if needed, is unclear in this subset, the best antithrombotic regime that should be implemented after the procedure remains unknown, conduction disturbances often limit the use of beta-blockers even when indicated for the treatment of persistent heart failure, and these factors altogether – so important for patient’s well-being – concur in a challenging scenario with several concomitant comorbidities and poly-medication [1]. Quality of life after the intervention is greatly influenced by the high rate of re-admissions (up to 44% within the first year post-TAVI) [5]. In most cases (~40%) readmissions are due to worsening heart failure that might be derived from persistent left ventricular dysfunction, significant para-valvular leak, concomitant mitral/tricuspid valvular disease, or even heart failure with preserved left ventricular ejection fraction [6,7]. Indication of renin-angiotensin inhibitors (RASi) is clear in most of these cases [8]. However, these drugs may pose a potential risk of hypotension/syncope episodes or renal failure decline, and even, as suggested by former studies in the cardiovascular surgery setting, an increased mortality [8]. Beyond these cases with clear indication due to persistent heart failure or left ventricular dysfunction, the RASTAVI study aimed to clarify the impact of RASi administration following TAVI in the prognosis of patients with preserved left ventricular ejection fraction and no other current indication for RASi [8]. The hypothesis was that RASi might help to reduce left ventricular hypertrophy as shown in some other alternative scenarios, through a more favorable left ventricular remodeling, and even increase the survival through a decrease in the amount of fibrosis [8], and, therefore, the risk of persistent heart failure. Surprisingly >40% of TAVI patients were not under any RASi. The results, after a matched comparison to avoid confounders, suggests that RASi in this context might be associated to a global cardiovascular protective effect (Figure 1), with a decrease in the rate of re-admissions, atrial fibrillation, stroke, and cardiovascular mortality at 3 years of follow up. This important finding is mechanistically reasonable since the central echocardiographic analysis demonstrated a decrease in both left ventricular size and hypertrophy. Interestingly, the global rate of mortality was not reduced, which can be probably explained through the common presence of severe comorbidities that caused > 50% of the deaths in the follow up as in most TAVI studies [1], thus highlighting the impact of other different features, like frailty and other geriatric syndromes, in terms of prognosis in these patients. The missing link between the improved remodeling and this cardiovascular protective effect found in the RASTAVI study is currently under investigation in the RASTAVI trial (NCT03201185) which will elucidate if the regression of myocardial fibrosis as determined by magnetic resonance– probably more extended in TAVI than in SAVR candidates given their advanced age – might bring about better survival [8]. The confirmation or refutation of this hypothesis is crucial in the post-TAVI pharmacological management - that currently remains highly empirical – given its important prognostic relevance.
